# Evaluation of the Antimicrobial Effect of Conventional and Nanosilver-Containing Varnishes on Oral Streptococci

**Published:** 2014-06

**Authors:** R. Haghgoo, H. Saderi, M. Eskandari, H. Haghshenas, MB. Rezvani

**Affiliations:** a Dept. of Pediatric Dentistry, Dental School, Shahed University, Tehran, Iran.; b Molecular Microbiology Research Center, Shahed University, Tehran, Iran.; c Dept. of Pediatric Dentistry, Dental School, Guilan University of Medical Sciences, Guilan, Iran.; d Dentist; e Dept. of Operative Dentistry, Dental School, Shahed University, Tehran, Iran.

**Keywords:** Antibacterial effect, Nanosilver, Oral streptococci

## Abstract

**Statement of the Problem:** Nanosilver particles have the potential to serve as a bactericidal agent because of the inherent antimicrobial influences of silver ion. The literature confirmed that specific micro-organisms, especially streptococci, have an important role as an etiological factor for caries.

**Purpose: **The aim of this study was to evaluate the antimicrobial effect of conventional and nanosilver-containing varnishes on oral streptococci.

**Materials and Method:** Pure cultivations of Streptococcus mutans and Streptococcus salivarius were prepared on blood agar media. Thereafter, 0.5 McFarland standard of recently grown bacteria in normal saline was prepared and the bacteria were cultivated monotonously on the culture medium surface by applying a swab. Different concentrations of nanosilver varnishes were prepared in the Mueller- Hinton broth medium in the test tubes and equal amounts of 0.5 McFarland suspension of all the tested bacteria were added separately to all test tubes. A tube without varnish was included as the control sample. The tubes were kept at 37°C for 24 hours, then cultured to determine the numbers of bacteria in each tube by counting colonies. The numbers of bacteria in tubes with varnish were compared to the numbers of bacteria in the tube without varnish. In the instance of observing any reduction in the growth, the minimum inhibitory concentration for growth in the tube with varnish was determined.

**Results:** Nanosilver varnish had an antimicrobial effect on S. mutans and S. salivarius.  S. salivarius was more susceptible than S. mutans to the nanosilver varnish.

**Conclusion:** Based on the results of this study, nanosilver varnishes can be used under amalgam restorations to reduce microbial population and subsequently preventing the recurrent caries.

## Introduction

Dental caries is one of the most important dilema in dentistry. Microorganisms, especially Streptococcus, serves as an imperative etiologic factor in dental caries. Extracellular polysaccharides of Streptococcus, in particular Streptococcus mutans, can induce and enhance the dental caries [1]. Nano-sized metal particles show specific biochemical and physical features [2-5] whereas bulk nanostructured materials show different behaviors [6-8]. In several studies that nanoparticles have been added to dental materials, it has been determined that nanopartices can modify dental materials’ mechanical properties [9-11]. In a study, nano siver was added to glass ionmer and its mechanical properties was evaluated. Results of this study showed that incorporation of nano silver to glass ionomer increased mechanical properties compared to originnal cement [9]. In another study, nano silver was added to conventional varnish and its microleakage was evaluated. The results showed that applying nanosilver to varnish reduced microleakage [10]. Silver nanoparticles are one of the products of nanotechnology. Antimicrobial effects of silver have been recognized for a long time. Silver, owing to its nanocrystal structure, has been noticeably concerned regarding its biological and antimicrobial values [12]. Silver nanoparticles have a greater surface area in comparison with the bulk silver particles which would increase their antimicrobial effect [13-17].

The antimicrobial effect of nanosilver has been investigated in several studies [13, 18-20]. In one study, it was specified that the antimicrobial effects of Vancomycin, Penicillin G, and Amoxicillin against Staphylococcus aureus have been increased in the presence of silver nanoparticles [18]. Another study surveyed the antimicrobial properties of silver nanoparticles and silver particles against Escherichia coli gram-negative bacteria and showed that the effects of silver nanoparticles were greater than the bulk silver particles [13]. An investigation enrolled by Hernandez- Sierra et al. reported that E. coli disappeared in the presence of nanosilver within several minutes [19].

Only one study has evaluated the antimicrobial  effect of nanosilver on S. mutans and its results showed that nanosilver had a high effect on S.mutans even in low concentrations [20].

To the best of our knowledge, there has been no research about the antimicrobial effect of nanosilver varnish on oral streptococci until the date of commencement of this study.The purpose of the current study was to evaluate the antimicrobial effect of nanosilver varnish on oral streptococci and subsequently, suggesting its application under amalgam restoration to reduce the numbers of bacteria.

## Materials and Method

Streptococcus mutans (PTCC1683) and Streptococcus salivarius (PTCC1448), in a lyophilized state, were purchased from the microbial collection of Iranian Scientific and Industrial Research Center. After adding sterile physiological serum (normal saline) to each vial of bacteria, they were cultured on blood agar surface in sterile conditions to grow the bacterial colonies. Then a homogenous suspension of bacteria colonies in 3 milliliters sterile vials( containing nutrient broth composition with 15% glycerol) was prepared and kept at -70^o^C in a refrigerator. It was necessary to provide the required amount of bacterial suspension from a 24-hour culture of frozen bacteria for each test episode. Therefore, immediately after filling each vial with water, 100 microliters of bacterial suspension were transferred to a tube containing 2 milliliters culture medium of streptococcus enrichment broth and kept at 37^o^C in an oven for 24 hours. Then, in the case of observing any turbidity in each inoculated tube, they were cultured on blood agar and selective streptococcal agar media and kept in an incubator for 24 hours to grow single bacterium colonies. Then to create a bacterial suspension turbidity of 0.5 McFarland;the colonies were removed by sterile phyldo platinum immediately before each test and were added to the tube containing Mueller- Hinton broth medium and stirred properly. All the employed culture media were prepared and kept according to the manufacturer’s (Merck Company; USA) instructions.

For preparing the varnishes with different concentrations of nanosilver; specific amounts of nanosilver based on their percentage in the final mixture (0.1, 0.5, 1, 2.5, and 5 percent) were weighed and added to the varnish solution. They were then properly stirred for 3 minutes by adopting a speed mixer machine.The final varnish concentration of the mixture was selected in a way that the inhibitory effect of the varnish itself on bacteria could not be observed.

Different concentrations of varnish were provided on Mueller–Hinton broth medium in test tubes and equal amounts of 0.5 McFarland suspension of all the tested bacteria were appended separately to all test tubes. A tube without varnish was considered as the control tube. The tubes were kept at 37◦C for 24 hours. Subsequently, each tube was cultured to define the amount of bacteria. The quantity of bacteria was determined by counting the colonies,. The numbers of bacteria in tubes that contained varnish was compared with the numbers of bacteria in the tube without varnish . In case of observing any reduction in growth, the minimum inhibitory concentration for growth in varnish was verified [21].

## Results

Following the inoculation of 8.4×107 S. mutans and 1.2×107 S. salivarius to each tube with different varnish concentrations and after 24 hours of incubation, bacterial growth inhibition was detected in tubes containing varnish.The number of bacteria were increasing in the control tubes without varnish (1.6×108 and 3.2×108 bacteria per milliliter, respectively). The minimum concentration of varnish for growth inhibition were determined to be 1/8 and 1/32 for S. mutans and S. salivarius, respectively .The minimum lethal concentration of S. mutans and S. salivarius were recorded as 1/4 and 1/16, respectively.

By inoculation of 3.6×106 S. mutans and 9.8×106 S. salivarius to each tube containing different concentrations of nanosilver and after 24 hours of incubation; the numbers of bacteria per millimeter in the 0.5 percent tube were determined to be 3.9×107 and 1.2×104, respectively, whereas in higher concentrations (1, 2.5, and 5 percent) there were no living bacteria. There were 8×107 and 2.4×108 bacteria, respectively in each millimeter of control tube. 

This experiment showed that nanosilver concentration of 1 percent or more can kill bacteria. In the 0.5 % nanosilver, S. mutans was still able to grow and after 24 hours, its number had reached up to ten times, while the numbers of S. salivarius were decreased. 

Therefore, the minimum nanosilver concentration for growth inhibition of S. mutans was more than 0.5 % and less than 1 %.

To establish the actual nanosilver concentration to inhibit growth of S. mutans; this experiment was conducted again with 0.5- to- 1% nanosilver for S. mutans. 

The results showed that in the concentrations  up to 0.7 %  nanosilver, the numbers of bacteria were equal to inoculated bacteria or more, however, they were decreased in concentration of 0.8% and reached to zero in the presence of nanosilver concentration of 1 %.

The results of the experiment in determining the effect of nanosilver-containing varnish on oral streptococci are illustrated in Table 1. Despite bacterial growth in the control tube, an inhibition in the increasing quantity of S. mutans was observed when exposed to varnishes containing 0.1% and 0.5 %nanosilver during 24 hours of incubation. The quantity of bacteria were decreased in the presence of varnishes containing 1% nanosilver or more, even though there was not a significant difference in the amount of bacterial reduction in these concentrations.

**Table 1:** Results of the experiment of determining the effect of nanosilver-containing varnish on oral streptococci (the numbers of inoculated S. mutans and S. salivarius in each tube were 8.9×107 and 1.8×107, respectively)

**Nanosilver concentration in varnish (%)**	**Bacteria per millimeter**	
	**S. mutans**	**S. salivarius**
0	4.2×109	8.3×108
0.1	3.5×107	8.4×105
0.5	1.2×107	7.3×104
1	8.2×106	3.6×103
2	6.4×106	4×102
5	2.3×106	0

For S. salivarius, the varnish containing 0.1 % nanosilver prompted bacterial reduction and varnishes containing higher concentrations showed a significant bacterial reduction. There was no bacterial growth with the 5 % nanosilver. Therefore, S. salivarius in comparison with S. mutans, had a higher susceptibility to antimicrobial and nanosilver varnishes.

## Discussion

Considering the vivid importance of oral streptococcus in caries etiology, prevention of the growth of these micro-organisms can be effective in prevention of dental caries.

Nanoparticles are defined as particulate dispersions or solid particles with a size of 10-100 nm [22]. These particles can be an optimal candidate for microbicides due to their efficacy in small doses, minimal toxicity, and minimal side effects [23]. Particle size and size distribution are the most important issues of nanoparticle systems [24].

The results of this study showed that nanosilver varnish had an antimicrobial effect on S. mutans and S. salivarius. Moreover, the susceptibility of S. salivarius to nanosilver varnish was more than that of S. mutans.

In the results of other studies, nanoparticles have demonstrated antibacterial activity ^25^ and antimicrobial products that contained nanoparticles, have been considered as the effective bactericidal materials [26].

The antibacterial and antiviral actions of silver, silver ions, and silver compounds have been reported in literature 26. Microorganisms are unlikely to become resistant against silver since they are susceptible to conventional antibiotics. Therefore, silver ions have been used as an antibacterial component in dental resin composites ^27^.

Results of some studies have demonstrated the bactericidal activity of silver nanoparticles ^14, 28^.

Sondi et al. concluded that silver nanoparticles would aggregate in the membrane of E. Coli, increase the permeability of the membrane and subsequently, damage the bacterial cell. Therefore, nanosilver can be used as a bactericidal material [13].

In another study, Morones et al. found that bactericidal features of nanosilver are dependent on the size of particles. The size of the nanoparticles must be between 1 to 10 nm to be directly exposed to bacteria [15].

According to the study of Lok et al., nanosilver with its own physicochemical characteristics, has similar antimicrobial activities to silver [28].

The conclusions of the aforementioned studies, similar to this study, support the antibacterial effect of nanosilver; however, this study has particularly evaluated the effect of nanosilver on oral streptococci.

Dental caries is a worldwide public health problem and S. mutans plays an important role in the etiology of caries. Some studies showed that the antimicrobial properties of silver nanoparticles are proposed  as an effective agent to diminish S. mutans [30-31].

Hernández-Sierra et al. demonstrated nanosilver’s antimicrobial effect against S. mutans in an *in vitro* study [29].

In a study by Shvero et al. [30], the anti-bacterial effects of a nanoparticle of polyethyleneimine against S. mutans and Enterococcus faecalis (E. faecalis) were evaluated. Their results revealed that the cement containing low concentrations of these particles has strong anti-bacterial properties for 14 days. Therefore, cement containing polyethyleneimine nanoparticles could help prevent dental caries and inflammation (pulpitis) [30]. They examined the effect of polyethyleneimine nanoparticles in the cement against S. mutans and E. faecalis whilst the aim of the present study was to investigate the antibacterial effect of nanosilver-containing varnish against S. mutans and S. salivarius.

Cavity varnish is normally used to make a barrier against the transmission of stimuli across cements or other restorative materials and to reduce the percolation of oral fluids to underneath dentin [31]. Nanosilver varnish can presumably reduce oral bacteria. However, no studies have been conducted in this field since the publishment of this survey.

Only in one study, Hernandez-Sierra and Ruiz studied the bactericidal and bacteriostatic effects of nanosilver. They compared the antibacterial effects of nanosilver with nanozinc oxide and gold nanoparticles. According to the results of their study, nanosilver in lower concentrations had a higher antibacterial effect on S. mutans compared with nanozinc oxide and gold nanoparticles. In their survey, the effects of nanosilver, nanozinc oxide, and gold nanoparticles on S. mutans were considered and the the high potential of these particles against S. mutans to eliminate the dental caries was concluded [19].

Some of these micro-organisms can produce enough acid to decalcify the dental structure. S. mutans is considered as a main micro-organism in cariogenesis. S. mutans and presumably S. sabrinus and Lactobacillus are human dental pathogens [24].

In the present study, the antibacterial effects of nanosilver-containing varnishes on oral micro organisms, namely S. mutans and S. sanguis, have been investigated. Future studies are suggested to evaluate this effect on other micro-organisms such as S. subrinus and Lactobacillus; which are also important in the occurrence and development of dental caries.

It is also suggested that other studies deliberate the micro leakage after implementing nanosilver varnishes.

## Conclusion

Based on the results of this study, nanosilver varnishes can be used under amalgam restorations to reduce microbial population and consequently; preventing the recurrent dental caries.
